# PELI2 inhibits colorectal cancer development through MAPK signaling pathway

**DOI:** 10.1186/s10020-025-01294-3

**Published:** 2025-06-12

**Authors:** Jialin Liu, Shengyun Hu, Liangbo Zhao, Yanmei Yang, Guanghua Wu, Yimeng Duan, Xinrui Ma, Peiwen Wang, Zhiyong Zhang, Hong Zong

**Affiliations:** 1 Department of Oncology, The First Affiliated Hospital of Zhengzhou University, Zhengzhou, 450001 China; 2 Department of Colorectal Surgery, The First Affiliated Hospital of Zhengzhou University, Zhengzhou, 450000 China; 3 School of Life Sciences, Zhengzhou University, Zhengzhou, 450001 China; 4 Academy of Medical Sciences, Zhengzhou University, Zhengzhou, 450001 China

**Keywords:** Colorectal cancer, PELI2, MAPK, Tumorigenesis

## Abstract

Colorectal cancer is one of the most common malignant tumors worldwide. Colorectal cancer has a poor survival rate because it tends to metastasise to the liver and other organs. PELI2, an E3 ubiquitin ligase, is down-expressed in a variety of tumors. However, the role of PELI2 in colorectal cancer has not been revealed. In the present study, we found that expression level of PELI2 were reduced significantly in colorectal tumors compared with normal tissues. Patients with low level of PELI2 expression tended to have poor prognosis. Moreover, PELI2 decreased the proliferation, migration and anti-apoptosis of colorectal cancer cells in vitro. We also constructed xenograft tumor model to verify colorectal tumor growth slowed down after PELI2 overexpression. Transcriptome analysis suggested that PELI2 suppressed colorectal cancer progression via the MAPK signaling pathway. In brief, our study shows that PELI2 inhibits colorectal cancer development by MAPK signaling pathway.

**Supplementary Information:**

The online version contains supplementary material available at 10.1186/s10020-025-01294-3.

## Introduction

Colorectal cancer (CRC) is the third most prevalent cancer in the world in terms of morbidity and mortality (Siegel et al. 2024). 20% colorectal cancer patients have already developed metastasis in liver, lung and other organs when first diagnosed (Biller and Schrag 2021). Currently, the main treatments for colorectal cancer include surgical resection, chemotherapy, targeted therapies and immunotherapy (Morris et al. 2023). However, survival rates are still not satisfactory due to high postoperative recurrence rates, suboptimal chemotherapy and limited therapeutic targets. The 5-year survival rate of CRC patients with metastasis is only 14% (Siegel et al. 2023). Metastasis occurs in approximately 50% of CRC patients and is the leading cause of death (Alhumaid et al. 2018). Consequently, there is an urgent need to explore the diagnosis and treatment targets in order to prolong the survival of CRC patients.

Ubiquitination, a critical post-translational modification, regulates diverse physiological processes (Swatek and Komander 2016). Among ubiquitination enzymes, E3 ligases determine substrate specificity (Morreale and Walden 2016) and has been regarded as the potential diagnostic and therapeutic target in cancer (Zhou and Sun 2021; Fujita et al. 2019). The human genome is now known to encode more than 600 E3 ligases (Popovic et al. 2014; Sampson et al. 2023). Pellino proteins are a group of highly conserved E3 ubiquitin ligases, including three members: Pellino 1 (Fei et al. 2024), Pellino 2 (PELI2) (Lin et al. 2008) and Pellino 3 (Jensen 2023; He et al. 2019). The role of PELI2 in cancer remains ambiguous due to the heterogeneity of cancer types. It has been proposed that PELI2 could promote the growth and metastasis of gastric cancer cells by regulating vascular endothelial growth factor C, considered as a sensitive predictive tool for lymphatic metastasis in gastric cancer (Zhang et al. 2019). Besides, PELI2 was significantly upregulated in B-cell precursor acute lymphoblastic leukemia cells and positive correlated with IL-7R (Xu et al. 2023). In addition, Kaplan-Meier survival analysis revealed that higher expression of PELI2 is associated with a better prognosis of myeloma (Masuda et al. 2022). However, the function of PELI2 in CRC have not been revealed.

In this study, we reported that PELI2 was down-regulated in CRC and inhibited tumor cell growth for the first time. This study systematically verified the function of PELI2 in CRC, providing a theoretical basis for the new strategy of targeted therapy for CRC (Dale et al. 2021).

## Results

### PELI2 is lowly expressed in CRC and correlated with CRC patient prognosis

**Fig. 1:**
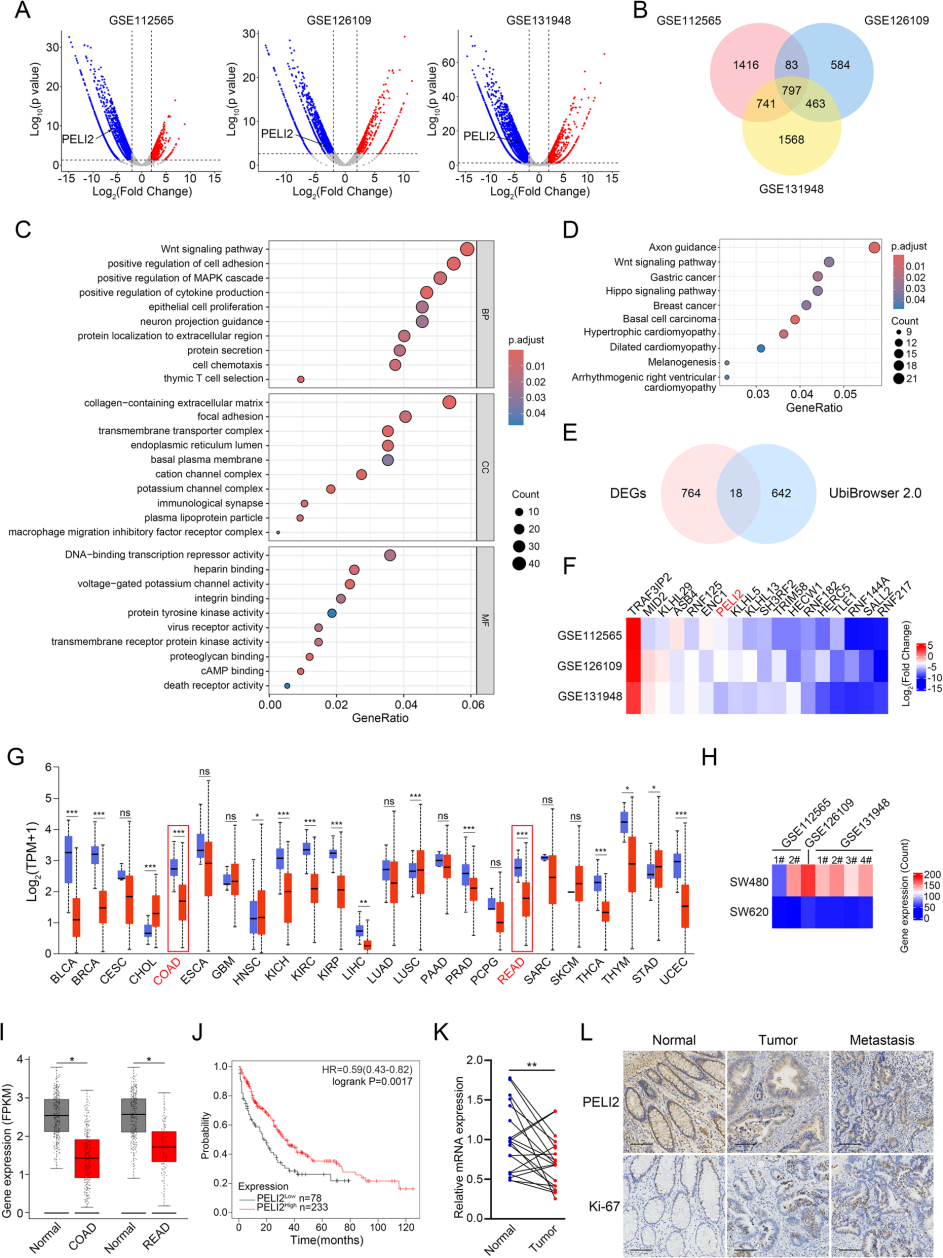
PELI2 is lowly expressed in colorectal cancer and correlated with colorectal cancer patient prognosis. **A** Volcanic maps of differential expressed genes in CRC in GSE112565, GSE126109 and GSE131948 data sets. The *p* value < 0.05 and |Log2(Fold Change)| > 2 were used as the selection criteria. **B** Venn plots of differential expressed genes in GSE112565, GSE126109 and GSE131948 data sets. **C**, **D** GO analysis (**C**) and KEGG analysis (**D**) of common differential expressed genes. **E** Venn plots of differential expressed genes and the E3 ubiquitin ligase database obtained from Ubibrowser v.2. **F** Heat map of 18 common differential expressed genes in (Fig. 1E). **G** Expression of PELI2 between tumors and normal samples according to UALCAN database. **H** Expression of PELI2 in three GSE datasets. **I** Expression of PELI2 was verified in COAD and READ according to GEPIA database. **J** Survival analysis was conducted between patients with high and low expression levels of PELI2 in COAD patients. **K** Expression of PELI2 in 20 pairs clinical CRC samples were detected by RT-qPCR. **L** Representative images of IHC for PELI2 in clinical CRC samples (scale bar 100 μm). All experiments were biological replicates and were repeated at least three times. Error bars showed standard error of the mean. *p < 0.05, **p < 0.01, ***p < 0.001

To explore potential therapy targets of CRC, we selected three CRC GSE datasets (GSE112565, GSE126109 and GSE131948) to analyze differential expressed genes (Fig. 1A). We identified 797 common differential expressed genes, including 99 upregulated genes and 683 downregulated genes (Fig. 1B). GO and KEGG analyze of differential expressed genes were performed. Differential expressed genes were mainly enriched including positive regulation of MAPK, epithelial cell proliferation and DNA-binding transcription repressor activity (Fig. 1C). In addition, differential expressed genes were mainly associated with KEGG pathways such as basal cell carcinoma, axonal conduction, and hypertrophic cardiomyopathy (Fig. 1D). We identified the intersection between the set of 660 human E3 ubiquitin ligases and differentially expressed genes in Fig. 1B and obtained 18 E3 ubiquitin ligases, which may be potential therapy targets of CRC (Fig. 1E). Among 18 ubiquitin ligases, SALL2, HERC5, RNF182, HECW1, TRIM58, SH3RF2, KLHL5, ENC1 and TRAF3IP2 have been shown to be involved in CRC development. Previous studies have shown that bioinformatics analysis indicates that RNF217, RNF144 A, TLE1, RNF125, ASB4, KLHL29, MID2 and KLHL13 are associated with CRC. However. the roles of PELI2 in CRC have not been revealed. Therefore, we chose PELI2 for next research (Fig. 1F). We found that the PELI2 was lowly expressed in a variety of cancers including colon cancer and rectal cancer (Fig. 1G). We provided specific expression levels of PELI2 in three GSE datasets (Fig. 1H). TCGA analysis showed that PELI2 was significantly low-expressed in colon and rectal cancer tissues (Fig. 1I). Survival analysis showed that low expression of PELI2 was associated with poor prognosis for patients with CRC (Fig. 1J). RT-qPCR and immunohistochemistry showed that PELI2 were reduced in patient colon cancer tissues and even lower in liver metastatic tissues (Fig. 1K and L). In conclusion, PELI2 expression is low in CRC and correlates with CRC patient prognosis.

### Knockdown of PELI2 increased the proliferation, migration and anti-apoptosis of CRC cells in vitro

**Fig. 2:**
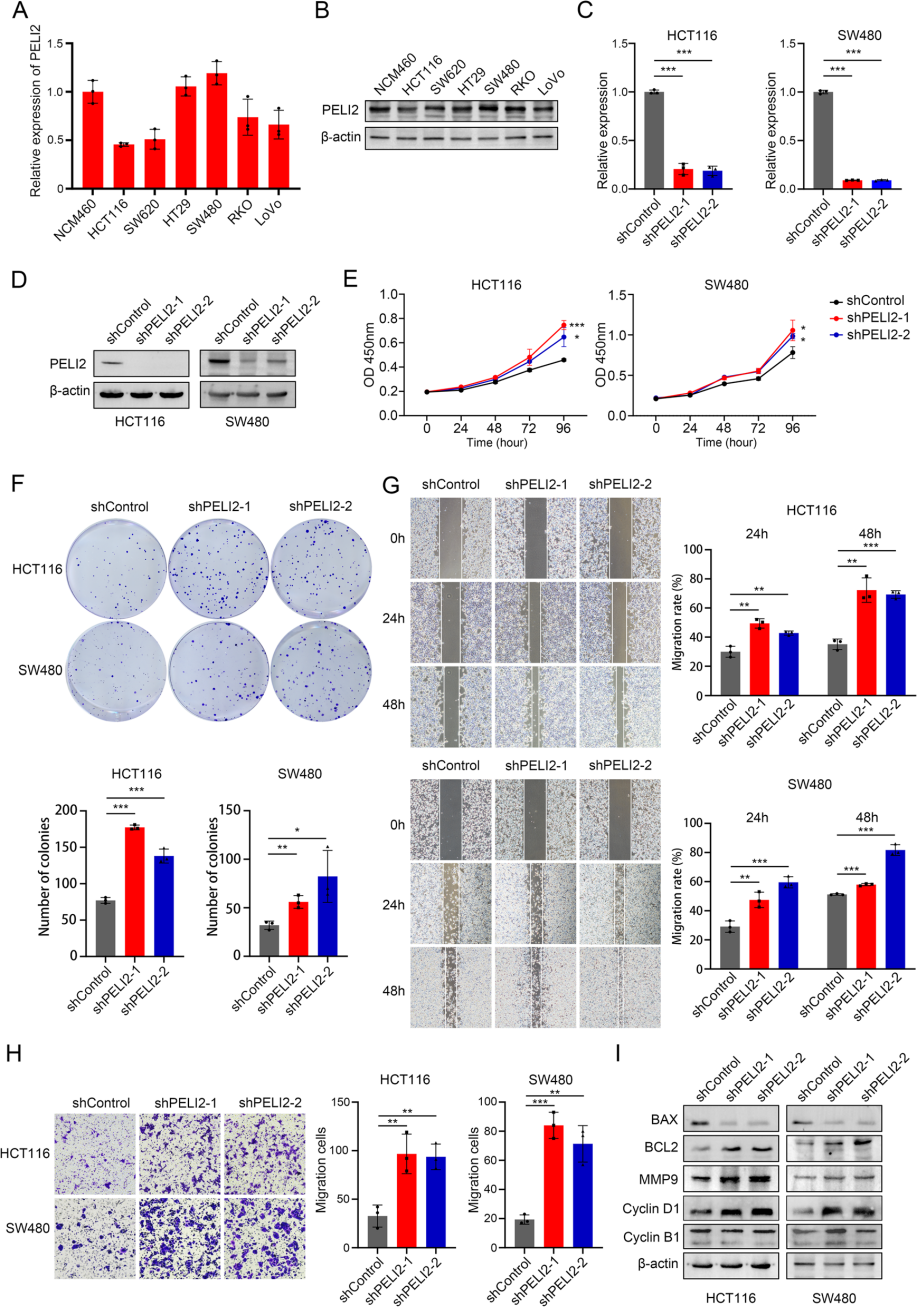
Knockdown of PELI2 increased the proliferation, migration and apoptosis of colorectal cancer cells in vitro. **A** RT-qPCR was used to determine the mRNA levels of PELI2 in six human CRC cell lines and the normal colon cell line NCM460. **B** Western blot was used to determine the expression levels of PELI2 in human CRC cell lines and the normal colon cell line NCM460. **C**, **D** The knockdown efficiency of PELI2 in HCT116 cells and SW480 cells was detected by RT-qPCR (**C**) and Western blot (**D**). **E** The cell viability increased after PELI2 knockdown in HCT116 cells and SW480 cells. **F** Colony Formation Assay of shControl and shPELI2 HCT116 and SW480 cells. Representative images are shown on the top, and statistical graph is shown on the down. **G** Scratch assay of shControl and shPELI2 HCT116 and SW480 cells. Representative images are shown on the left, and statistical graph at 24 h and 48 h is shown on the right. **H** Transwell assay of shControl and shPELI2 HCT116 and SW480 cells. Representative images are shown on the left, and statistical graph is shown on the right. **I** Protein expression levels of BAX, BCL2, MMP9, Cyclin D1, and Cyclin B1 were verified in shControl and shPELI2 HCT116 and SW480 cells. All experiments were biological replicates and were repeated at least three times. Error bars showed standard error of the mean. *p < 0.05, **p < 0.01, ***p < 0.001

In order to explore the function of PELI2, we detected PELI2 expression in six CRC cell lines and normal colon tissue cells (Fig. 2A and B). RT-qPCR and Western blot showed that PELI2 was lower in HCT116 cell line and higher in SW480 cell line. Then we established PELI2 stable knockdown HCT116 cells and SW480 cells. The mRNA expression (Fig. 2C) and protein expression (Fig. 2D) of shPELI2 were significantly decreased in both HCT116 cells and SW480 cells. Compared with shControl, shPELI2 increased the growth rate (Fig. 2E) and colony formation ability (Fig. 2F) of HCT116 cells and SW480 cells. To further investigate the effect of PELI2 on cell migration, we performed scratch assay and transwell assay. We found migration rate was significantly promoted in shPELI2 HCT116 cells and SW480 cells (Fig. 2G and H). In addition, we examined the protein expression levels of BAX, BCL2, MMP9, Cyclin D1, and Cyclin B1. Western blot demonstrated that BAX was significantly downregulated, whereas BCL2, MMP9, and Cyclin D1 were markedly upregulated in shPELI2 HCT116 cells and SW480 cells (Fig. 2I). Notably, Cyclin B1 expression remained unaltered under PELI2 knockdown conditions. These findings collectively indicate that PELI2 knockdown enhances proliferation, migration, and anti-apoptotic potential in CRC cells in vitro.

### Overexpression of PELI2 decreased the proliferation, migration and anti-apoptosis of CRC cells in vitro

**Fig. 3:**
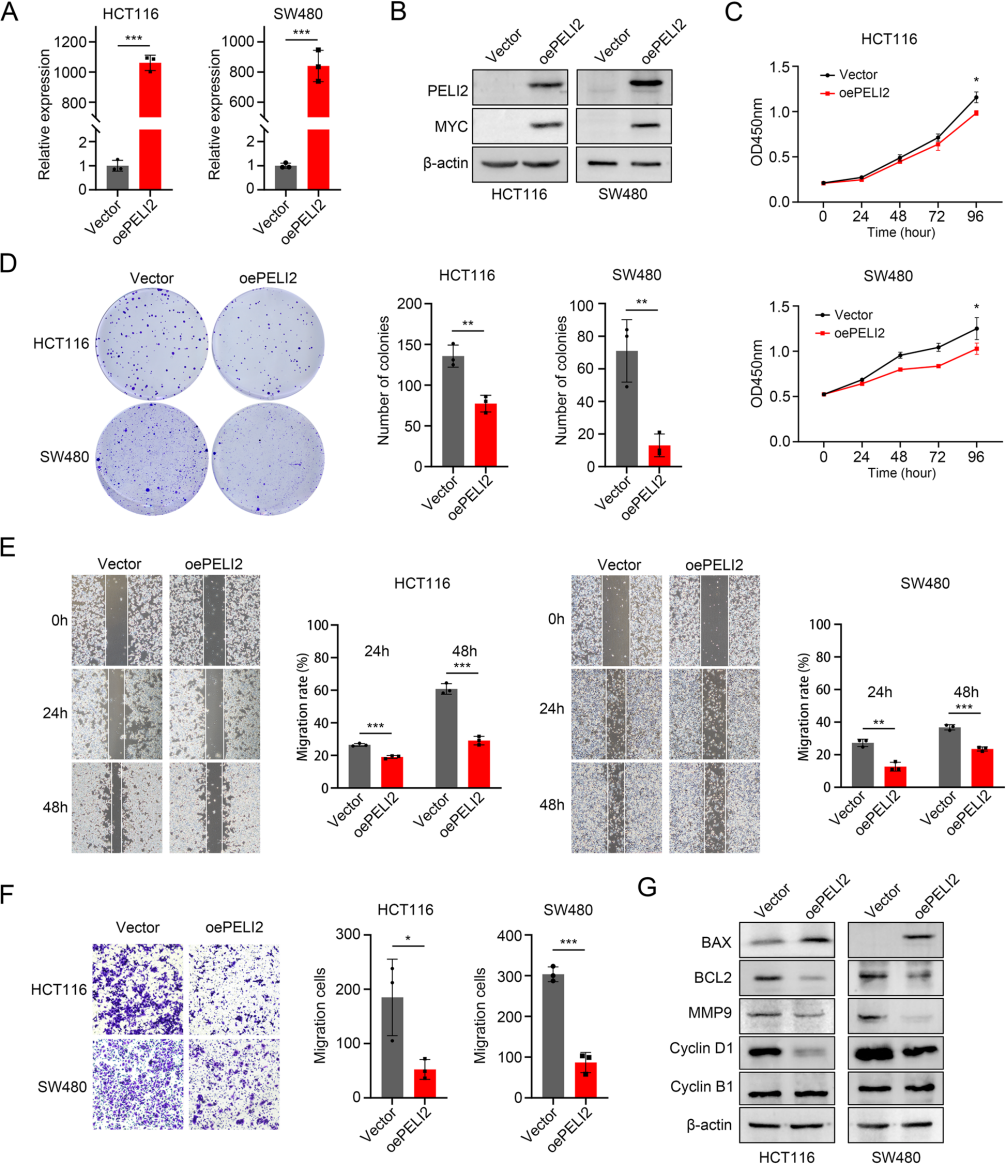
Overexpression of PELI2 decreased the proliferation, migration and apoptosis of colorectal cancer cells in vitro. **A**, **B** Overexpression efficiency of PELI2 in HCT116 cells and SW480 cells was detected by RT-qPCR (**A**) and Western blot (**B**). **C** The cell viability reduced after PELI2 overexpression in HCT116 cells and SW480 cells. **D** Colony Formation Assay of Vector and oePELI2 HCT116 and SW480 cells. Representative images are shown on the left, and statistical graph is shown on the right. **E** Scratch assay of Vector and oePELI2 HCT116 and SW480 cells. Representative images are shown on the left, and statistical graph at 24 h and 48 h is shown on the right. **F** Transwell assay of Vector and oePELI2 HCT116 and SW480 cells. Representative images are shown on the left, and statistical graph is shown on the right. **G** Protein expression levels of BAX, BCL2, MMP9, Cyclin D1, and Cyclin B1 were verified in Vector and oePELI2 HCT116 and SW480 cells by Western blot. All experiments were biological replicates and were repeated at least three times. Error bars showed standard error of the mean. *p < 0.05, **p < 0.01, ***p < 0.001

We established MYC-PELI2 stable overexpression HCT116 cells and SW480 cells. mRNA expression (Fig. 3A) and protein expression (Fig. 3B) was significantly increased in oePELI2 HCT116 cells and SW480 cells. Compared with Vector, PELI2 overexpression decreased the growth rate (Fig. 3C) and colony formation ability (Fig. 3D) of HCT116 cells and SW480 cells. Scratch assay revealed that the migration rate was significantly descended in oePELI2 HCT116 cells and SW480 cells (Fig. 3E). We also found that PELI2 overexpression restrained the migration of HCT116 cells and SW480 cells with Transwell assays (Fig. 3F). Moreover, we examined the protein expression levels of BAX, BCL2, MMP9, Cyclin D1, and Cyclin B1. Western blot analysis revealed that BAX was significantly upregulated, while BCL2, MMP9, and Cyclin D1 were markedly downregulated in oePELI2 HCT116 and SW480 cells (Fig. 3G). Notably, Cyclin B1 expression remained unchanged under these conditions. These results collectively demonstrate that PELI2 overexpression suppresses proliferation, migration, and anti-apoptotic capacity in CRC cells in vitro.

### Overexpression of PELI2 represses colorectal tumor growth in vivo

**Fig. 4:**
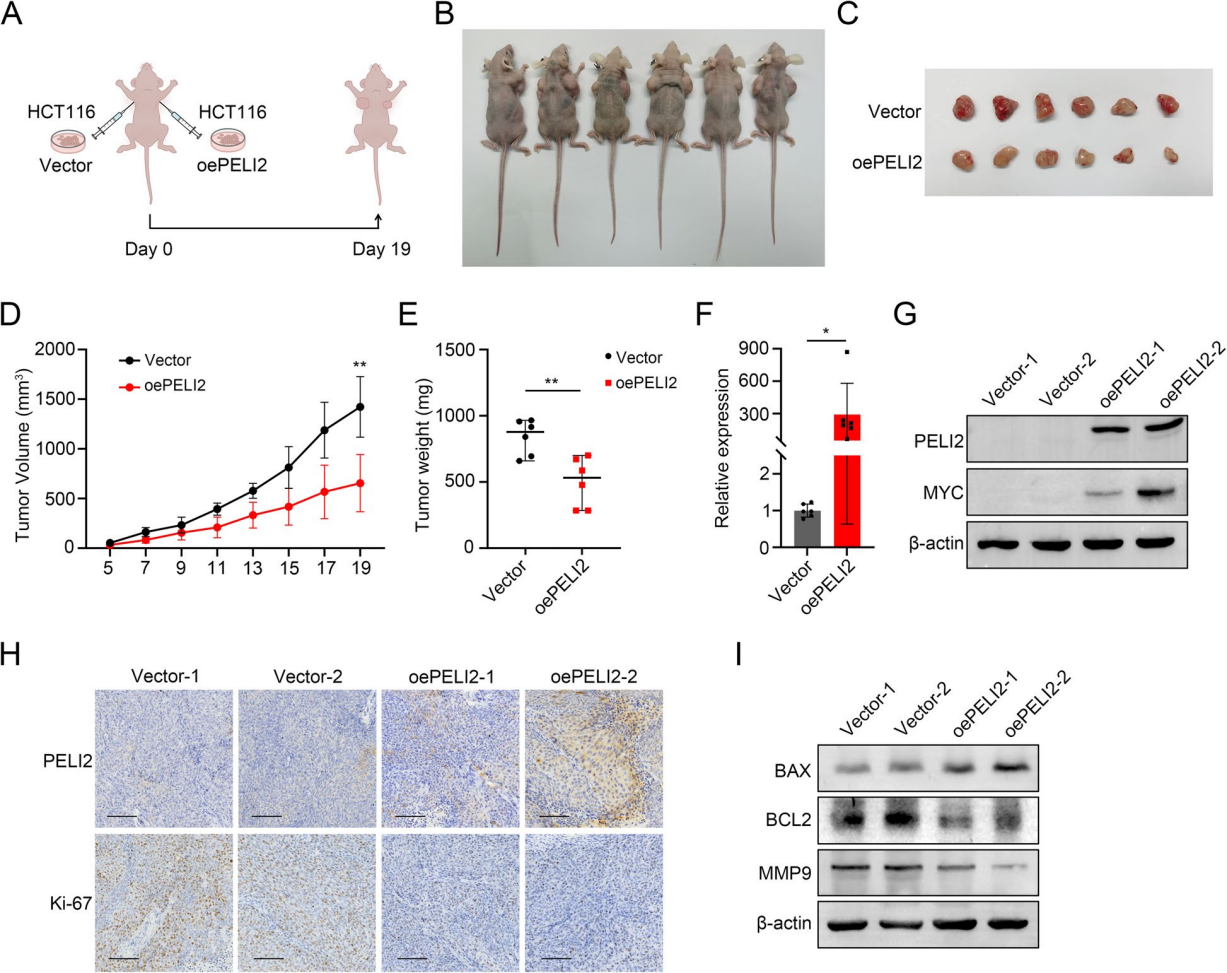
Overexpression of PELI2 represses colorectal tumor growth in vivo. **A** The schematic diagram of establishing subcutaneous xenograft tumor model in nude mice. **B** Final presentation of subcutaneous xenograft tumor in nude mice (*n* = 6). **C** Dissected tumors from subcutaneous xenograft tumor model in nude mice were shown. **D** Tumor volumes were measured every 2 days and growth curves were plotted. **E** The tumor weights of the two groups were measured. **F**, **G** RT-qPCR (**F**) and Western blot (**G**) was performed to evaluate the mRNA and expression levels of PELI2 in collected tumor tissues. **H** Representative images of IHC for Ki67 and PELI2 in indicated tumor tissues (scale bar 100 μm). **I** Protein expression levels of BAX, BCL2 and MMP9 were verified in tumor tissues. All experiments were biological replicates and were repeated at least three times. Error bars showed standard error of the mean. *p < 0.05, **p < 0.01, ***p < 0.001

In order to verify the role of PELI2 on CRC tumor growth in vivo, we constructed nude mouse xenograft tumor model (Fig. 4A). 5×10^6^ Vector and oePELI2 HCT116 cells were injected subcutaneously into both sides of nude mice. After nineteen days, we found oePELI2 HCT116 cells grew smaller tumor compared to Vector HCT116 cells (Fig. 4B-E). We further extracted RNA and protein from tumors, and found that PELI2 expression levels of oePELI2 group tumors were significantly higher compared to Vector group tumors (Fig. 4F and G). Immunohistochemical staining showed that the PELI2 expression in oePELI2 group tumors was significantly higher than Vector group tumors, and Ki-67 staining confirmed that oePELI2 could reduce proliferation of tumor cells in vivo (Fig. 4H). Besides, we detected protein expression levels of BAX, BCL2 and MMP9. Western blot showed that BAX was observably increased while BCL2 and MMP9 were reduced in oePELI2 group tumors (Fig. 4I). In conclusion, overexpression of PELI2 can inhibit tumor formation ability of CRC cells in vivo.

### PELI2 restrains CRC development through MAPK signaling pathway

**Fig. 5:**
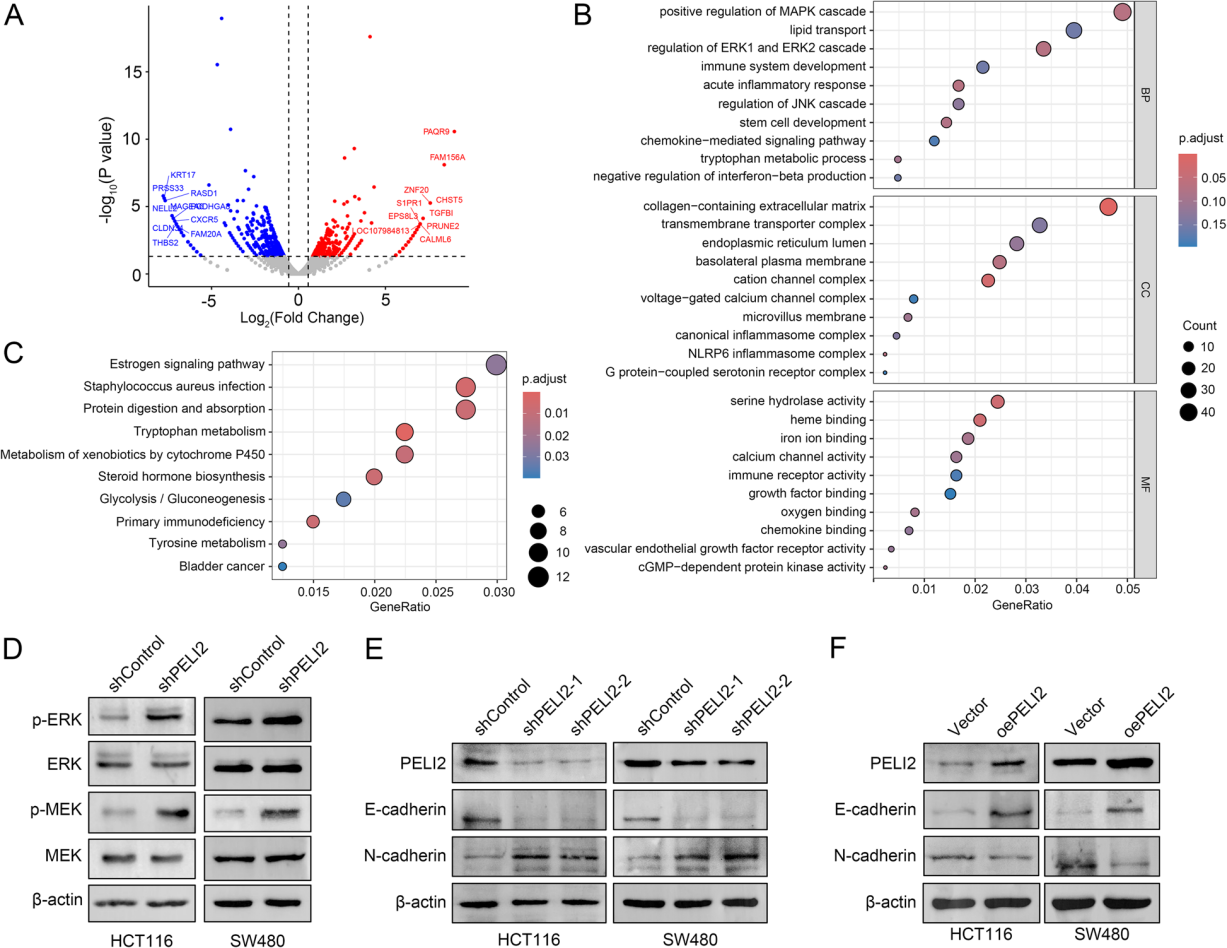
PELI2 restrains colorectal cancer development through MAPK signaling pathway. **A** Volcanic map shows differential expressed genes derived from RNA-seq data in shControl and shPELI2 HCT116 cells. The *p* value < 0.05 and |logFC| > 0.56 were used as the selection criteria. **B**, **C** GO analysis (**B**) and KEGG analysis (**C**) of differential expressed genes derived from RNA-seq data of shControl and shPELI2 HCT116 cells. **D** Protein expression levels of p-ERK, ERK, p-MEK and MEK were verified in shControl and shPELI2 HCT116 and SW480 cells. **E** Protein expression levels of E-cadherin and N-cadherin in shControl and shPELI2 HCT116 and SW480 cells. **F** Protein expression levels of E-cadherin and N-cadherin in Vector and oePELI2 HCT116 and SW480 cells

To elucidate specific mechanism by which PELI2 inhibits CRC development, we used transcriptome sequencing technology (RNA-Seq) to analyze gene expression between shControl and shPELI2 HCT116 cells. 782 differential expressed genes were found in shControl and shPELI2 group, including 304 up-regulated genes and 478 down-regulated genes (Fig. 5A). Go analysis of these genes revealed that they were mainly enriched in MAPK signaling pathway (Fig. 5B). In addition, KEGG analysis showed that differential expressed genes were mainly associated with estrogen signaling pathway, Staphylococcus aureus infection and protein digestion and absorption (Fig. 5C). Western blot analysis further verified that PELI2 knockdown in both HCT116 and SW480 cells significantly activated phosphorylation of ERK and MEK, key effectors of the MAPK signaling pathway (Fig. 5D). To address the potential link between PELI2-mediated MAPK regulation and epithelial-mesenchymal transition (EMT) (Ren et al. 2023), we examined EMT markers in these cells. Notably, PELI2 knockdown led to a marked reduction in E-cadherin and concomitant upregulation of N-cadherin, whereas PELI2 overexpression reversed this trend, restoring E-cadherin expression and suppressing N-cadherin levels in both cell lines (Fig. 5E, F). These results suggest that PELI2 modulates EMT progression through MAPK signaling. Collectively, our data demonstrate that PELI2 inhibits CRC development by suppressing the MAPK pathway and its downstream EMT program.

## Discussion

CRC is frequently diagnosed at advanced stages due to asymptomatic progression and insufficient early screening (Qu et al. 2022), with 15–30% of patients presenting metastasis at diagnosis and 20%−50% patients with initially localised disease will develop metastasis (Cervantes et al. 2023). The unclear molecular mechanisms driving CRC pathogenesis hinder effective therapies, underscoring the urgent need to identify promising biomarkers and therapeutic targets. In this study, we identify PELI2, an E3 ubiquitin ligase, as a potential tumor suppressor in CRC. Our data suggest that PELI2 expression is significantly downregulated in CRC tissues, and its deficiency correlates with poor prognosis. Functionally, PELI2 depletion enhances CRC cell proliferation, migration, and anti-apoptotic capacity in vitro, while overexpression suppresses these phenotypes. Xenograft models further indicate that PELI2 overexpression inhibits tumor growth in vivo. Through transcriptomic analyses, we propose that PELI2 primarily restrains CRC progression via suppression of the MAPK signaling pathway and its downstream EMT program, with potential contributions to cell cycle regulation, such as Cyclin D1 modulation. However, the precise mechanisms and additional pathways influenced by PELI2 remain to be fully elucidated.

The Pellino protein family, first identified in Drosophila melanogaster (Zhang and Li 2022), comprises three highly conserved members (PELI1-3) in mammals (Jensen 2023). While Pellino proteins are best known for their roles in immunity (Humphries and Moynagh 2015) and inflammation (Zhang and Li 2022; Moynagh 2014), recent studies reveal their divergent functions in cancer. For example, PELI1 activates the PI3 K/Akt/GSK3β signaling pathway, leading to poor prognosis of patients (Fei et al. 2024), and PELI3 promotes colorectal carcinogenesis through TLR4-mediated inflammation (Kim et al. 2023). However, the physiological functions of PELI2 remain poorly understood. Previous research on PELI2 focused on inflammatory responses (Humphries et al. 2018), and and some studies have also linked PELI2 to leukaemia (Modarres et al. 2021), gastric cancer (Zhang et al. 2019), and multiple myeloma (Masuda et al. 2022). Notably, TCGA data and our clinical analyses reveal consistent PELI2 downregulation in CRC, where low expression predicts poor prognosis. Our data suggest that PELI2 may act through suppression of the MAPK pathway in CRC, as evidenced by ERK/MEK activation upon PELI2 knockdown and subsequent EMT induction. This contrast between its pro-tumorigenic roles in other cancers and tumor-suppressive function in CRC underscores the need for tissue-specific mechanistic studies.

The MAPK pathway drives CRC progression not only through proliferation signals (Ma et al. 2021) but also by orchestrating cell cycle (Scheiblecker et al. 2020) and EMT programs (Xu et al. 2018). ERK promots cell invasion and migration by up-regulating Matrix metalloproteinases (de Almeida et al. 2022) and EMT (Jinesh and Brohl 2022) molecules. It has been shown that monobutyl phthalate-mediated down-regulation of PELI2 increased ubiquitination of IRAK1, which activated the MAPK/JNK signaling pathway and led to proliferation of normal mouse testicular Sertoli cells (Ma et al. 2020). Our data reveal that PELI2 depletion upregulates Cyclin D1—a master regulator of G1/S transition (Bertoli et al. 2013)—without altering Cyclin B1 levels, suggesting selective disruption of early cell cycle checkpoints. Cyclin D1 overexpression is a hallmark of CRC aggressiveness, uncontrolled proliferation (Yang et al. 2020) and chemoresistance (Wang et al. 2019). Concurrently, PELI2 loss induces EMT, marked by E-cadherin downregulation and N-cadherin upregulation. This phenotypic shift is likely mediated by MAPK-dependent activation of EMT transcription factors (Olea-Flores et al. 2019). While our findings highlight PELI2’s role in cell cycle and EMT regulation, other pathways may also contribute to its tumor-suppressive effects. Beyond transcriptional regulation, bioinformatic analysis of TCGA data revealed a potential epigenetic layer of PELI2 suppression in CRC. Hypermethylation of the PELI2 promoter correlated significantly with poorer overall survival (*p*<0.01), suggesting DNA methylation as a plausible mechanism for its downregulation. Although these findings are exploratory, they align with emerging evidence.

In conclusion, this study is the first to reveal the tumor-suppressive role of PELI2 in CRC. And its downregulation is significantly associated with poor prognosis. Through in vitro and in vivo experiments, we demonstrated that PELI2 effectively suppresses CRC cell proliferation, migration, and anti-apoptotic capacity by inhibiting the MAPK signaling pathway and its downstream EMT program. However, the precise molecular targets of PELI2 and its potential crosstalk with other oncogenic pathways remain to be elucidated. Future studies should focus on identifying PELI2-specific ubiquitination substrates via proteomic approaches (Kim et al. 2011) and validating its therapeutic potential in larger clinical cohorts. Additionally, developing small-molecule agonists targeting the PELI2-MAPK axis may offer novel strategies for advanced CRC treatment (Liu et al. 2018).

## Materials and methods

### Antibodies

The following antibodies were used in our study: Anti-PELI2 Polyclonal antibody (Cat# 16097-1-AP, Proteintech), Anti-β-actin antibody (Cat# A5441, Sigma), Anti-Myc Antibody (Cat# sc-40, Santa Cruz), Anti-BAX Polyclonal antibody (Cat# 50599-2-Ig, Proteintech), Anti-BCL2 Polyclonal antibody (Cat# 26593-1-AP, Proteintech), Anti-MMP9 Polyclonal antibody (Cat# 10375-2-AP, Proteintech), Anti-Cyclin D1 Polyclonal antibody (Cat# 26939-1-AP, Proteintech), Anti-Cyclin B1 Polyclonal antibody (Cat# 55004-1-AP, Proteintech), Anti-MEK1/2 Polyclonal antibody (Cat# 11049-1-AP, Proteintech), Anti-Phospho-MEK1/2 (Ser217/221) antibody (Cat# 9121, CST), Anti-p44/42 MAPK (Erk1/2) (137 F5) Rabbit mAb (Cat# 4695, CST), Anti-Phospho-p44/42 MAPK (Erk1/2) (Thr202/Tyr204) antibody (Cat# 9101, CST),Anti-E-cadherin antibody (Cat# TA0131,Abmart), Anti-N Cadherin antibody (Cat# T55015,Abmart), Anti-mouse HRP (Cat# ZB-2305, Zsbio), Anti-rabbit HRP (Cat# ZB-2301, Zsbio).

### Immunohistochemistry

Tissue paraffin blocks were sliced into 4–5 μm and then dewaxed and rehydrated, treated with hydrogen peroxide at room temperature to inhibit endogenous peroxidase, naturally restored to room temperature after antigen repair. After being blocked with donkey serum for 30 min, the tissue sections were incubated with primary antibodies at 4 °C overnight, followed by incubation with a peroxidase-labeled secondary antibody for 30 min at room temperature. After diaminobenzidine (DAB) reaction was developed, the slides were counterstained with hematoxylin.

### Cell culture

The human normal colonic epithelial cell line NCM460 was purchased from BLUEFBIO (Cat# BFN608006385). The human cell line 293 T and the human CRC tumor cell lines were provided by Pingping Zhu (School of Life Sciences, Zhengzhou University). Human colorectal cancer cell lines HCT116, HT29, SW480, SW620, LoVo, RKO and human normal colorectal epithelial cell NCM460 were cultured in DMEM and RPMI-1640 medium supplemented with 10% fetal bovine serum (Cat# S711-001S, Lonsera) and 1% penicillin-streptomycin (Cat# C0222, Beyotime) at 37 °C in humidified 5% CO_2_ atmosphere.

### Vector construction

We looked for targets on Merck public website (https://www.sigmaaldrich.cn/CN/zh) and designed shRNA with a fixed structure (target sequences: shPELI2-1: GCCTCATGGAACTCATGCATT; shPELI2-2: GAACCTTACACAGCACGGATA). For construction of knockdown vectors, we connected shRNA to pLKO.1 puro vector (Cat# 10878, Addgene). For construction of overexpression vectors, we cloned PELI2 coding sequence into pLVX-IRES-ZsGreen1 (Cat# 632187, Takara). The recombined plasmid was extracted using endotoxin-free plasmid extraction kit and sequenced correctly.

### Lentiviral infection

The knockdown plasmid was co-transfected into HEK293 T cells with packaging plasmids psPAX2 (Cat# 12260, Addgene) and pMD2.G (Cat# 12259, Addgene). HEK293 T cells would be 70–80% confluent at the time of transfection. Incubate for 8 hours after adding the above two helper plasmids and then replace the medium. Virus particles were collected after 48 hours, filtered by 0.45 μm syringe filter and added to HCT116 or SW480 with 50% fusion. Finally, replace the medium after 8 hours.

### Quantitative real-time PCR and RNA-sequencing

All RNA was extracted from cells and inverted into cDNA with Hifair® AdvanceFast One-step RT-gDNA Digestion SuperMix (Cat# R223-01, Vazyme). Choose human β-actin gene as internal reference gene for relative quantification, The primer sequences are as follows: PELI2-F: GGATATGTTTCAGGTGGGCAGA, PELI2-R: TGGAAGAGTCAAATCCGGCG, ACTB-F: GCCGACAGGATGCAGAAGGAGATCA, ACTB-R: AAGCATTTGCGGTGGACGATGGA. The relative expression of genes in each sample was calculated according to the relative expression formula 2^-ΔΔCt^. RNA library preparation, quality assessment, and sequencing were conducted by BGI Genomics (Shenzhen, China). For RNA-seq data visualization in the IGV genome browser, raw sequencing reads were processed using the bamCoverage tool from deepTools (v3.5.1) with read-per-genomic-content (RPGC) normalization to generate bigWig files. Gene Ontology (GO) and Kyoto Encyclopedia of Genes and Genomes (KEGG) pathway enrichment analyses were performed using the R package clusterProfiler (v4.6.2). Normalized enrichment scores (NES) and empirical p-values were calculated under default parameters, followed by false discovery rate (FDR) adjustment using the Benjamini–Hochberg method.

### Western blot

RIPA (Cat# P0013B, Beyotime) and PMSF (Cat# P0100, Solarbio) were added to cells, and protein was determined using BCA protein quantification kit (Cat# P0012, Beyotime). Proteins were separated on 10% SDS-polyacrylamide gels and transferred to PVDF membranes (Cat# IPVH00010, Millipore). The membranes were then blocked in 5% nonfat milk at 4 °C overnight, and incubated with the primary antibodies at room temperature for 2 hours. The horseradish peroxidase-conjugated secondary antibodies (1:5000) were used to incubate the membranes. β-actin was used as internal reference. Finally, ECL detection kit (Cat# P0018S, Beyotime) was used to detect protein.

### CCK-8 assay

Cells were seeded into 96-well plates at the initial density of 2000 cells/well and cultured in CO_2_ cell incubator. Cell proliferation was estimated by the CCK-8 (Cat# K1018, APExBio), which was added to each well (10 μl/well) at the time points of 24 h, 48 h, 72 h and 96 h. After incubation at 37 °C for 40 min, absorbance value at 450 nm (OD450) was measured with microplate reader.

### Colony formation assay

HCT116 and SW480 cells were isolated and seeded in 6-well plates with 500 cells per well. After being cultured for 7 days, cells were fixed with 4% paraformaldehyde before being subjected to crystal violet staining. The clone number was measured using a scanner and processed by Image J.

### Scratch assay

HCT116 and SW480 cells were seeded in 6-well plates with 70–80% confluent, culturing in DMEM medium supplemented with 0.5% fetal bovine serum (FBS) and 1% penicillin-streptomycin sulfate. Confluent cells were scraped using 10 μL pipette tip. After 24 h and 48 h, the cells migrated to the wound and the scratched area were examined using inverted microscope. The migration rate was obtained by the formula: (Width0 h−Width 24 hor48 h)/Width0 h.

### Transwell assay

The Transwell permeable supports (Cat# 3422, Corning) were placed into the 24-well plate, and added 700 μL DMEM containing 10% FBS to the lower chamber and 250 μL cell fluid without FBS to the upper chamber. After 48 h, the cells were fixed with 4% paraformaldehyde and stained with crystal violet. The migrated cells could be counted using microscope.

### Clinical specimens

Clinical colorectal cancer samples were obtained from The First Affiliated Hospital of Zhengzhou University. All patients provided informed consent and were pathologically and clinically confirmed to have colorectal cancer. All experiments were approved by the Ethics Committee of Zhengzhou University.

### Animal models

All animal procedures were conducted in accordance with protocols approved by the Institutional Animal Care and Use Committee (IACUC) of Zhengzhou University. Female BALB/c nude mice (4–5 weeks old) were procured from SPF Biotechnology Co., Ltd. (Beijing, China). The sample size for this study was determined empirically without formal statistical power calculations. Additionally, investigators were not blinded during data collection or analysis. In order to establish colorectal cancer xenograft model, HCT116 cells (5×10^6^) were injected subcutaneously into both sides of all BALB/c nude mice. The length and width of tumors were measured with caliper and tumor volume (mm^3^) was calculated with the formula: tumor volume (mm^3^) = longer diameter×shorter diameter^2^/2. Then mice were sacrificed and the tumor tissues were isolated and frozen in liquid nitrogen or fixed in formalin immediately.

### Statistical analysis

We explored PELI2 expression in pan-cancer using UALCAN (http://ualcan.path.uab.edu/). R (version 4.3.2) and GraphPad Prism 9.5.1 software were used for statistical analysis. The unpaired Student’s t-test was used to compare significant differences. All results data were showed as mean value ± SD. P-value lower than 0.05 were considered to indicate statistical significance. *p<0.05, ** *p*<0.01 and *** *p*<0.001.

## Supplementary Information

Supplementary Material 1.

## Data Availability

No datasets were generated or analysed during the current study.
